# A C-Terminal Protease-Resistant Prion Fragment Distinguishes Ovine “CH1641-Like” Scrapie from Bovine Classical and L-Type BSE in Ovine Transgenic Mice

**DOI:** 10.1371/journal.ppat.1000137

**Published:** 2008-08-29

**Authors:** Thierry Baron, Anna Bencsik, Johann Vulin, Anne-Gaëlle Biacabe, Eric Morignat, Jérémy Verchere, Dominique Betemps

**Affiliations:** Agence Française de Sécurité Sanitaire des Aliments–Lyon, Unité ATNC, Lyon, France; University of Edinburgh, United Kingdom

## Abstract

The protease-resistant prion protein (PrP^res^) of a few natural scrapie isolates identified in sheep, reminiscent of the experimental isolate CH1641 derived from a British natural scrapie case, showed partial molecular similarities to ovine bovine spongiform encephalopathy (BSE). Recent discovery of an atypical form of BSE in cattle, L-type BSE or BASE, suggests that also this form of BSE might have been transmitted to sheep. We studied by Western blot the molecular features of PrP^res^ in four “CH1641-like” natural scrapie isolates after transmission in an ovine transgenic model (TgOvPrP4), to see if “CH1641-like” isolates might be linked to L-type BSE. We found less diglycosylated PrP^res^ than in classical BSE, but similar glycoform proportions and apparent molecular masses of the usual PrP^res^ form (PrP^res^ #1) to L-type BSE. However, the “CH1641-like” isolates differed from both L-type and classical BSE by an abundant, C-terminally cleaved PrP^res^ product (PrP^res^ #2) specifically recognised by a C-terminal antibody (SAF84). Differential immunoprecipitation of PrP^res^ #1 and PrP^res^ #2 resulted in enrichment in PrP^res^ #2, and demonstrated the presence of mono- and diglycosylated PrP^res^ products. PrP^res^ #2 could not be obtained from several experimental scrapie sources (SSBP1, 79A, Chandler, C506M3) in TgOvPrP4 mice, but was identified in the 87V scrapie strain and, in lower and variable proportions, in 5 of 5 natural scrapie isolates with different molecular features to CH1641. PrP^res^ #2 identification provides an additional method for the molecular discrimination of prion strains, and demonstrates differences between “CH1641-like” ovine scrapie and bovine L-type BSE transmitted in an ovine transgenic mouse model.

## Author Summary

The origin of the transmissible agent involved in the food-borne epidemic of bovine spongiform encephalopathy (BSE) remains a mystery. It has recently been proposed that this could have been the result of the recycling of an atypical, more probably sporadic, form of BSE (called bovine amyloidotic spongiform encephalopathy, or L-type BSE) in an intermediate host, such as sheep. In this study we analyzed the molecular features of the disease-associated protease-resistant prion protein (PrP^res^) found in the brain of transgenic mice overexpressing the ovine prion protein after experimental infection with prions from bovine classical and L-type BSEs or from ovine scrapie. Scrapie cases included rare “CH1641-like” isolates, which share some PrP^res^ molecular features with classical BSE and L-type BSE. Scrapie isolates induced in transgenic mouse brains the production of a C-terminally cleaved form of PrP^res^, which was particularly abundant from “CH1641-like” cases. In contrast, this C-terminal prion protein product was undetectable in ovine transgenic mice infected with bovine prions from both classical and L-type BSE. These findings add a novel approach for the discrimination of prions that may help to understand their possible changes during cross-species transmissions.

## Introduction

Prion diseases such as Creutzfeldt-Jakob disease (CJD) in humans, scrapie in sheep and goats and bovine spongiform encephalopathy (BSE) in cattle are tightly associated with the accumulation of an abnormal form of a host-encoded cellular prion protein (PrP C) in infected tissues [1]. The biochemical properties of this disease-associated form of the protein (PrPd), which include insolubility in non-denaturing detergents and partial resistance to degradation by proteases, differ from those of the normal form. Whereas the normal protein is fully sensitive to proteases, the abnormal prion protein is only partly degraded (PrP^res^) due to removal of the amino-terminal end. In most cases, a large protease-resistant C-terminal core fragment is identified which has a gel mobility of ∼19–21 kDa in its unglycosylated form. However, in some prion diseases, such as some cases of human Creutzfeldt-Jakob disease [2] or the H-type atypical form of BSE [3], a much smaller C-terminal PrP^res^ product has also been reported.

A typical molecular signature of the BSE agent has been identified by PrP^res^ Western blot analysis, which allows such methods to be used to identify the possible presence of BSE in sheep or goats [4]–[10]. The origin of the BSE agent in cattle is still unknown, and its possible reservoir has not yet been identified. A few isolates of TSEs were described in sheep that showed partial similarities with experimental ovine BSE, with a lower molecular mass of unglycosylated PrP^res^ than in most scrapie cases, as found in ovine BSE. However the very high proportions of diglycosylated PrP^res^ found in ovine BSE were not generally apparent in such isolates. This was first demonstrated in the CH1641 experimental scrapie isolate [11],[12], then in a few natural scrapie cases in Great Britain and France [13],[14]. Bioassays performed in wild-type mice to identify prion strains from TSE isolates were reported to identify the biological signature of the BSE agent [15]–[18], but the CH1641 source failed to transmit the disease to such mice [11],[12]. Both CH1641 and “CH1641-like” natural isolates were however transmitted in an ovine transgenic mouse model (TgOvPrP4), showing similar PrP^res^ molecular features in both transgenic mice and sheep, i.e. a low apparent molecular of unglycosyslated PrP^res^ (referred as l-type PrP^res^) [19],[20]. In some of the cases this could be not the unique molecular phenotype identified in all scrapie-infected mice, with some of the mice also showing PrP^res^ with a higher apparent molecular mass (h-type PrP^res^) [20].

Deviant phenotypes of BSE have recently been reported in cattle however (H and L-types, based on the PrP^res^ features in cattle brain) [21]–[24]. Bioassays in wild-type and transgenic mice showed that these were consistent with the presence of two distinct strains, both differing from the single classical BSE strain involved in the food-borne BSE epidemic [23], [25]–[27]. Thus, the possible transmission of such forms of BSE in other species such as small ruminants also needs to be considered. Recently the hypothesis has been raised that the classical BSE epidemic might have originated from the recycling of one of these atypical forms of BSE (L-type BSE) after a first cross-species transmission, possibly in sheep [27],[28]. Recent studies of a transmissible mink encephalopathy (TME) isolate in TgOvPrP4 mice also showed similar phenotypic features to those of L-type BSE, suggesting a possible cross-species transmission of L-type BSE by oral route [29]. Given their unusual molecular properties, “CH1641-like” or CH1641 isolates might be the result of a transmission of L-type BSE to sheep or might represent similar isolates occurring in sheep.

In this study we compared the PrP^res^ molecular features of a series of natural “CH1641-like” and experimental CH1641 scrapie isolates, with those of classical and L-type BSE, after transmission to TgOvPrP4 ovine transgenic mice. We demonstrated the abundance of a C-terminal PrP^res^ fragment (PrP^res^ #2), which distinguished these ovine scrapie isolates from both bovine classical and L-type BSEs after transmission in a common ovine trangenic mouse model.

## Results

### “CH1641-like” isolates and L-type BSE share similar Western blot features to the usual PrP^res^ form (PrP^res^ #1) in TgOvPrP4 mice

**Figure 1:**
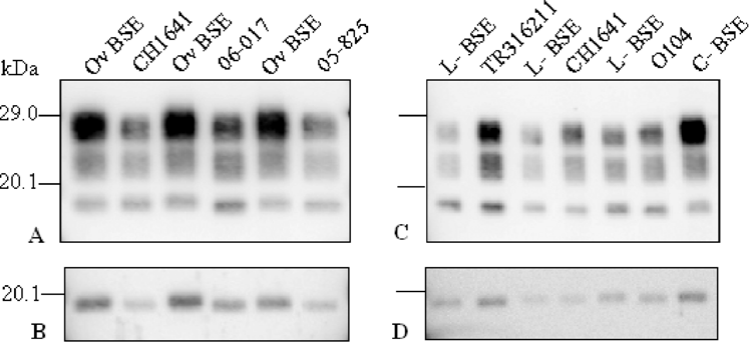
Western blot analysis of PrP^res^ from “CH1641-like” isolates in TgOvPrP4 mice detected by Bar233 monoclonal antibody. CH1641 and natural “CH1641-like” (06-017, 05-825, TR316211, and O104) scrapie isolates are compared with experimental ovine BSE (Ov BSE), L-type (L-BSE), or classical (C-BSE) BSE in cattle. (A) and (B) show results in TgOvPrP4 mice at first passage and (C) and (D) at second passage. PrP^res^ was analysed before ([A] and [C]) or after ([B] and [D]) PNGase deglycosylation.

**Figure 2:**
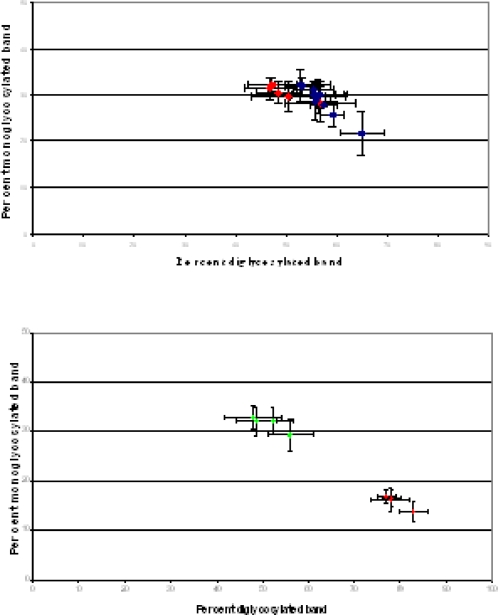
Glycoform ratios of PrP^res^ from “CH1641-like” isolates in TgOvPrP4 mice. Glycoform ratios of PrP^res^ in individual mice inoculated with ovine or bovine isolates (first passage) are shown in (A) and (B), respectively. In (A), CH1641 is shown in red, and natural isolates 06-017 and 05-0825 in blue. In (B), Classical and L-type BSE are shown in red and green, respectively. PrP^res^ was detected by Bar233 antibody.

We compared the PrP^res^ Western blot profiles, after transmission in TgOvPrP4 ovine transgenic mice, of two recently identified natural sheep TSE isolates (05-825 and 06-017) that showed PrP^res^ molecular features comparable to the experimental CH1641 scrapie isolate, i.e., a low apparent molecular mass (l-type) close to that found in ovine BSE. When the Bar233 antibody was used to detect the usual form of PrP^res^ (PrP^res^ #1), the molecular features, i.e. the apparent molecular masses of the three PrP^res^ glycoforms and the glycoforms proportions, were similar in all PrP^res^ positive mice in both experimental groups (Figure 1A). The glycoform proportions in each of the natural 4 “CH1641-like” isolates (or in CH1641) were significantly different from those of classical BSE (*p*<0.0001 for all 15 tests), with essentially lower levels of diglycosylated PrP^res^ than in classical BSE (Figures 1A and 2A). Lane by lane comparisons revealed a slightly lower apparent molecular mass of unglycosylated PrP^res^ in mice infected with scrapie rather than ovine BSE, and also after PNGase deglycosylation (Figure 1B). Nevertheless, these differences (0–0.3 kDa) remained within the range of the possible variations of an individual sample in a given Western blot experiment. These molecular features were similar to those found in TgOvPrP4 mice infected with two other previously described “CH1641-like” natural scrapie isolates, at first or second passages (Figure 1C and 1D), in all (TR316211 isolate) or in some (O104 isolate) of the mice [20].

In contrast, the PrP^res^ #1 molecular features in TgOvPrP4 mice infected with “CH1641-like” isolates did not differ from those found in mice infected with L-type BSE. The low apparent molecular mass of unglycosylated PrP^res^ was similar to that found in mice infected with classical BSE (Figure 1C), and also after PNGase deglycosylation (Figure 1D). Glycoform proportions did not differ significantly between any of the natural “CH1641-like” isolates (or CH1641) and L-type BSE with all cases showing lower levels of diglycosylated PrP^res^ than in classical BSE (Figures 1C and 2B) (*p*>0.30 for all tests except the one comparing the monoglycosylated PrP^res^ band in TR316211-infected mice for which *p* = 0.07).

Low apparent molecular masses of PrP^res^ were consistently associated with strongly reduced labeling by P4 monoclonal antibody (data not shown).

### A C-terminal PrP^res^ fragment (PrP^res^ #2) is detected in ovine transgenic mice infected with scrapie isolates but not with L-type or classical BSE

**Figure 3:**
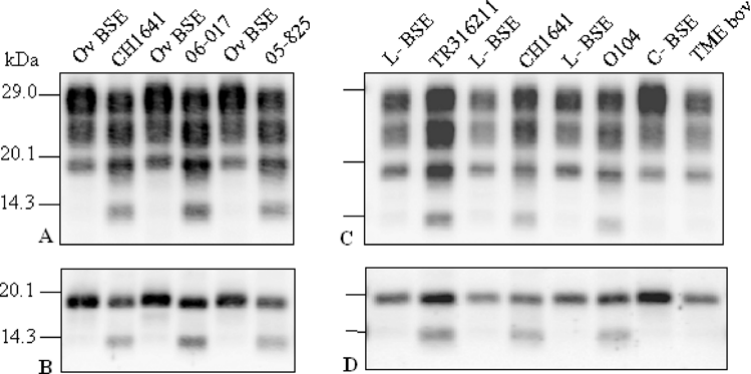
Western blot analysis of PrP^res^ from “CH1641-like” isolates in TgOvPrP4 mice detected by SAF84 monoclonal antibody. CH1641 and natural “CH1641-like” (06-017, 05-825, TR316211, and O104) scrapie isolates are compared with ovine BSE (Ov BSE), L-type (L-BSE), or classical (C-BSE) natural BSE in cattle and transmissible mink encephalopathy experimentally transmitted to cattle (TME bov) [29]. (A) and (B) show results in TgOvPrP4 mice at first passage and (C) and (D) at second passage. PrP^res^ was analysed before ([A] and [C]) or after ([B] and [D]) PNGase deglycosylation.

We then used SAF84 for PrP^res^ detection, that identified an additional band at ∼14 kDa (PrP^res^ #2) in TgOvPrP4 mice infected with the four natural “CH1641-like” isolates and with the experimental CH1641 isolate (Figure 3A and 3C). This was associated with lighter, more diffuse labeling below the well defined ∼19 kDa unglycosylated PrP^res^ #1 band, consistent with the presence of a monoglycosylated form derived from the ∼14 kDa PrP^res^ product. This PrP^res^ #2 fragment was not detected in mice infected with classical BSE transmitted to sheep or with L-type BSE in cattle (Figure 3A and 3C). The existence of two distinct PrP^res^ fragments of ∼19 and ∼14 kDa detected with SAF84 antibody only in the “CH1641-like” isolates was also demonstrated after deglycosylation by PNGase treatment (Figure 3B and 3D). Comparison of PrP^res^ profiles between TgOvPrP4 mice and sheep or cattle (Figure S1) indicate that TgOvPrP4 faithfully reproduced PrP^res^ features of ruminants following transmission of scrapie or BSE, including regarding the presence of PrP^res^ #2 in “CH1641-like” scrapie but not in BSE.

The ∼14 kDa band was not detected in TgOvPrP4 mice that had been infected with an isolate from cattle experimentally infected with transmissible mink encephalopathy (TME) (Figure 3), previously demonstrated to have phenotypic features similar to L-type BSE in TgOvPrP4 mice (PrP^res^ of low apparent molecular mass) [29].

**Figure 4:**
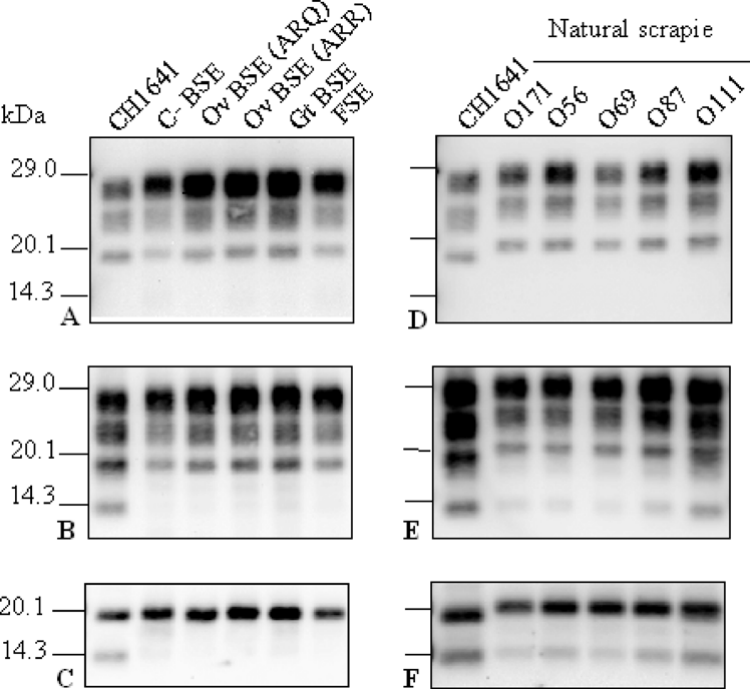
Western blot analysis of PrP^res^ from BSE sources and from “non-CH1641-like” isolates in TgOvPrP4 mice. BSE sources shown in (A–C) include BSE from cattle (lane 2), sheep homozygous for the A_136_R_154_Q_171_ (lane 3) or A_136_R_154_R_171_ (lane 4) *prnp* allele, goat (lane 5), and cheetah (lane 6). “Non-CH1641-like” natural scrapie isolates include O171, O59, O69, O87, and O111 scrapie isolates. PrP^res^ in TgOvPrP4 mice (first passage) was analysed before ([A,B,D,E]) or after ([C] and [F]) PNGase deglycosylation. PrP^res^ was detected by Bar233 antibody in (A) and (D) and by SAF 84 antibody in (B), (C), (E), and (F).

We analyzed PrP^res^ in TgOvPrP4 mice infected from six different BSE sources including (i) 3 natural isolates from cattle, goat, and a cheetah with feline spongiform encephalopathy (FSE) and (ii) 3 experimental sources from sheep (homozygous either for A_136_R_154_Q_71_ or A_136_R_154_R_171_ *prnp* allele) or from C57Bl/6 wild-type mice. All these BSE sources showed a similar PrP^res^ profile with low apparent molecular mass (∼19 kDa), close to that found in CH1641-infected mice, but with higher levels of diglycosylated PrP^res^, after the use of both Bar233 and SAF84 antibodies (Figures 4A, 4B, and 6). In contrast to the CH1641 source, none of them showed detectable levels of the ∼14 kDa PrP^res^ fragment with SAF84 antibody, even after PNGase deglycosylation (Figure 4B and 4C).

However, a ∼14 kDa band was also detected in mice infected with 5 natural scrapie isolates (Figure 4E), otherwise characterized by a higher apparent molecular mass of PrP^res^ #1 compared to CH1641 (Figure 4D) or to ovine BSE, although this ∼14 kDa band was clearly less intense than from “CH1641-like” isolates.

### The C-terminal PrP^res^ fragment (PrP^res^ #2) is preferentially associated with PrP^res^ of low apparent molecular mass

**Figure 5:**
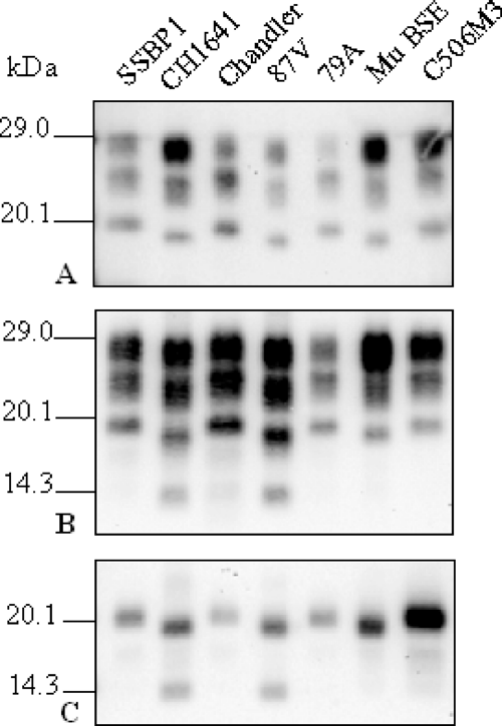
Western blot analysis of PrP^res^ from experimental scrapie and BSE sources in TgOvPrP4 mice. Scrapie sources include SSBP1 and CH1641 experimental scrapie isolates and Chandler, 87V, 79A, and C506M3 scrapie strains in TgOvPrP4 mice (second passage). BSE is derived from C57Bl/6 mice infected with classical BSE (Mu BSE) (second passage in TgOvPrP4 mice). PrP^res^ was analysed before (A, B) or after (C) PNGase deglycosylation and detected using Bar233 (A) or SAF84 (B, C).

We then evaluated the presence of the C-terminally cleaved PrP^res^ fragment detected by SAF84 antibody in the experimental sources that had been adapted to TgOvPrP4 mice. (1) Among the two experimental scrapie isolates, unlike CH1641, this PrP^res^ fragment was not detected from the SSBP/1 isolate (Figure 5). (2) From experimental strains derived from mouse-adapted scrapie or BSE strains, it was only detected in the 87V strain of scrapie which is also characterized by a low apparent molecular mass of the unglycosylated PrP^res^ #1 form, similar to that found in BSE, but not in the three other scrapie strains (C506M3, Chandler and 79A) otherwise characterized by a high molecular mass of unglycosylated PrP^res^ (Figure 5).

**Figure 6:**
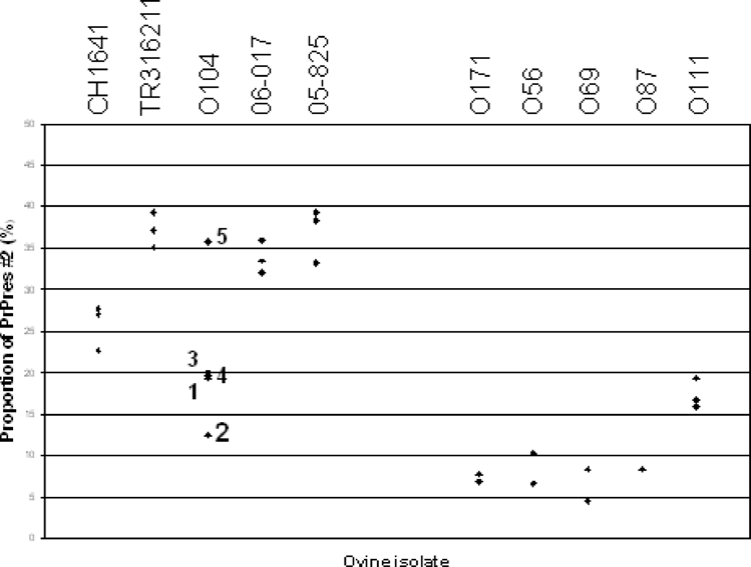
Proportions of PrP^res^ #2 from scrapie sources transmitted to TgOvPrP4 mice. Proportions of PrP^res^ #2 were evaluated by repeated Western blot analyses of the samples after PNGase deglycosylation and detection using SAF84 antibody. In the case of O104-infected mice, the mice are numbered as indicated in Figure 7 showing Western blot results.

In mice infected with natural scrapie isolates and CH641, the respective proportions of the ∼14 and ∼19 kDa bands, as observed after PNGase deglycosylation, were quantified and the ratios of PrP^res^ #2/PrP^res^ #1 determined (Figure 6). The mean proportions of PrP^res^ #2 in the “CH1641-like” isolates (3–5 mice analysed per experimental group) represented 22.7% to 39.3% of the total signal, except for the O104 isolate for which these proportions were smaller (12.4%–20%) in 4 of the 5 mice analysed. The proportions of PrP^res^ #2 in mice infected with the other scrapie isolates (“non CH1641-like”) (1–3 mice analysed per experimental group), were below 10% in most mice, except those infected with O111 (15.9%–19.4%). Statistical analyses of the data confirmed the significantly higher proportions of PrP^res^ #2 in mice infected with “CH1641-like” isolates (or CH1641) compared with other scrapie isolates (*p*<0.0001), as well as the significantly higher proportions of PrP^res^ #2 in the O111 isolate within the “non CH1641-like” isolates (*p* = 0.02). No significant differences in these proportions of PrP^res^ #2 were found between the natural “CH1641-like” isolates and the experimental CH1641 isolate (*p* = 0.42).

**Figure 7:**
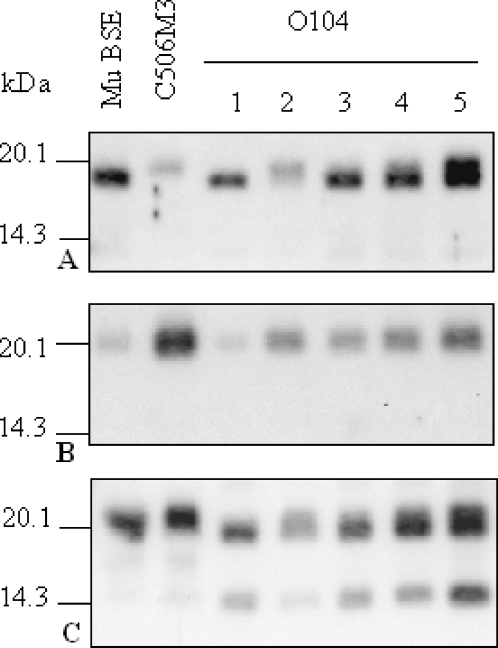
Western blot analysis of PrP^res^ from O104 natural scrapie isolate in TgOvPrP4 mice. PrP^res^ was analysed in TgOvPrP4 mice (first passage) by Western blot after PNGase deglycosylation and detected using Bar233, P4, and SAF84 monoclonal antibodies in (A), (B), and (C), respectively. PrP^res^ from individual mice infected with O104 isolate (first passage) is shown, with BSE derived from BSE-infected C57Bl/6 mice (Mu BSE) and C506M3 scrapie controls in TgOvPrP4 mice (second passage). O104-infected mice are numbered as indicated in Figure 5 showing the proportions of PrP^res^ #2.

Transmission studies of the O104 isolate showed the presence of a mixture of two distinct PrP^res^ phenotypes, with either high (h-type) or low (l-type) apparent molecular masses of unglycosylated PrP^res^, in variable proportions in each individual mouse, as shown using Bar233 detection after PNGase treatment (Figure 7A) [20]. The l-type PrP^res^, compared to the h-type, is only faintly labeled by the P4 antibody, but a P4-labelled PrP^res^ sub-population that migrates similarly to the h-type PrP^res^ can be identified in mice with l-type PrP^res^ (Figure 7B). When the SAF84 antibody was used (Figure 7C), the C-terminal PrP^res^ fragment was detected in all the O104 infected mice, but the lowest proportion (12.4%) (Figure 6) was found in the sole mouse that showed the most important proportions of h-type PrP^res^ (lanes 4 in Figure 7). These data are also consistent with a preferential association of PrP^res^ #2 with PrP^res^ #1 of low apparent molecular mass (l-type) in this scrapie isolate.

### Differential immunoprecipitation shows PrP^res^ #2 as a glycosylated PrP^res^ fragment

**Figure 8:**
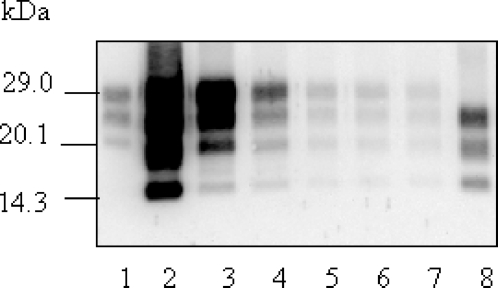
Enrichment of PrP^res^ #2 by differential immunoprecipitation. Western blot analysis of PrP^res^ from TgOvPrP4 infected with a “CH1641-like” isolate (TR316211). PrP^res^ released from beads after each capture cycle is shown for 5 successive cycles using Sha31-coated beads (lanes 3 to 7, respectively), then from the following capture cycle using SAF84-coated beads (lane 8). PrP^res^ controls from C506M3 strain and TR316211 are shown on lanes 1 and 2, respectively. PrP^res^ was detected using SAF84 antibody that recognizes both PrP^res^ #1 and PrP^res^ #2 forms.

Immunoprecipitation experiments were carried out to enrich the PrP^res^ #2 form in the samples and characterize it. Successive rounds of immunoprecipitation on magnetic beads coated with Sha31 N-terminal antibody that only recognizes PrP^res^ #1, allowed progressive depletion of the PrP^res^ #1 in the samples (Figure 8). After 7 rounds of immunoprecipitation, PrP^res^ #1 becomes only barely detectable. Immunoprecipitation was then performed using the C-terminal SAF84 antibody that recognizes both PrP^res^ #1 and PrP^res^ #2. Three bands were detected at ∼22, 18, and 14 kDa, showing that PrP^res^ #2, previously identified as an unglycosylated ∼14 kDa band, is also isolated from the mouse brains in monoglycosylated and diglycosylated forms.

The presence of this three band PrP^res^ #2 form was confirmed by differential immunoprecipitation in CH1641, “CH1641-like” isolates, and 87V but also in the O111 “non-CH1641-like” scrapie isolate. It could not be detected in mice infected with ovine BSE, L-type BSE or cattle experimentally infected with transmissible mink encephalopathy.

### Neuropathological investigations of “CH1641-like” isolates in TgOvPrP4 mice

The neuropathological analyses of the first passage experiments indicated comparable distribution of disease-associated prion protein in the brain of the transgenic mice among the “CH1641-like” sheep scrapie group (Figure S2). This was particularly clear for the 05-825 and 06-017 isolates that resulted in similar intensity of pathological PrP accumulation (Figure S2C and S2D). Overall, these data were also not dissimilar from those already described for the first passage of L-type BSE [29]. In both “CH1641-like” scrapie and L-type BSE, the florid plaque type of PrPsc deposition reported in this transgenic mouse line infected with classical BSE was never observed. However it is possible to underline some clear distinctive features such as a difference in the cortex targeting that was less intense compared to L-type BSE, even in the most severely affected cases (05-825 and 06-017 isolates) (Figure S2E). Remarkably the types of PrP^d^ deposition were also different; in the “CH1641-like” sheep scrapie group the deposition of pathological PrP was fine granular compared to L-type BSE in which plaque-like deposition were sometimes noticeable. Also, in the mesencephalon (raphe dorsalis), the deposition was intraneuronal for the “CH1641-like” sheep scrapie group but not in the brain of mice infected with L-type BSE. These data thus indicate some differences in the biological features of “CH1641-like” isolates, not only with classical BSE, but also with L-type BSE.

## Discussion

This study describes the molecular analyses of PrP^res^ after transmission into TgOvPrP4 ovine transgenic mice from 4 natural ovine scrapie isolates whose PrP^res^ features in sheep were similar to those previously described for the experimental CH1641 scrapie isolate [12]. Two of these previously unreported isolates (05-825 and 06-017) behaved as previously described for CH1641 and another natural isolate (TR316211) during the first passage in TgOvPrP4 mice, showing low molecular mass PrP^res^ (l-type PrP^res^) in all mice [19],[20]. In contrast, all the TgOvPrP4 mice receiving 5 natural scrapie isolates characterized by high PrP^res^ molecular masses (h-type PrP^res^) in the sheep brain, showed PrP^res^ of high molecular mass. Detailed analyses showed, as previously described in the CH1641 isolate in sheep [9] and in TgOvPrP4 mice [19], a slightly lower PrP^res^ molecular mass in TgOvPrP4 mice from the “CH1641-like” isolates than from ovine BSE, although the resolution of small gels made discrimination difficult. Our results are quite consistent with previous studies of the CH1641 isolate by the immunohistochemical “peptide mapping” method, which revealed that PrP^d^ in the CH1641 isolate was truncated further upstream in the N terminus than from experimental BSE [30]. The biochemical PrP^res^ features of these scrapie isolates differ from BSE mainly in their moderately high proportions of di-glycosylated PrP^res^ (50%–60%), whereas ovine BSE is characterized by higher proportions of di-glycosylated PrP^res^ [4],[9],[12]. Molecular discrimination of strains based on the relative proportions of glycoforms is however less reliable than that of PrP^res^ molecular masses, given the large measurement variations and poor standardization of analytical methods [9], [10], [31]–[33]. Furthermore glycoforms proportions of BSE in sheep have only been determined from a very limited number of sources. A recent study of classical BSE in cattle showed large individual variations (∼20%) in the proportions of di-glycosylated PrP^res^ [34].

The question of a possible transmission of BSE in small ruminants now needs to be re-examined considering the recent identification of atypical cases of BSE (H-type or L-type) in cattle [21]–[24]. Recent studies have indeed hypothesized that cross-species transmission of such rare atypical cases could be at the origin of the BSE epidemic in cattle [27],[28],[35]. The first experimental support for this hypothesis was obtained following the discovery of a BSE-like phenotype in mice following transmission of L-type BSE in wild-type mice (C57Bl, SJL) [27] or in an ovine transgenic (tg338) mouse line [28]. However, unlike tg338, which expressed 8- to 10-fold levels of V_136_ R_154_ Q_171_ ovine PrP, the phenotype of the L-type BSE remained distinct from classical BSE during at least two passages in TgOvPrP4 mice that expressed 2- to 4-fold levels of the A_136_ R_154_ Q_171_ ovine PrP [29]. It is noteworthy that, in cattle, the essential difference between L-type BSE and classical BSE is the slightly lower apparent molecular mass and the lower proportions of diglycosylated PrP^res^ [22]–[24], reminiscent of the differences between CH1641 and classical BSE experimentally transmitted to sheep [9],[12],[30]. The phenotypic features of L-type BSE have not yet been reported in sheep. In this study we showed that the PrP^res^ molecular masses and glycoform proportions between “CH1641-like” scrapie isolates and L-type BSE transmitted into TgOvPrP4 mice were indistinguishable, in addition to survival periods in the same range at second passage.

However our study revealed that a highly sensitive C-terminal antibody (SAF84) recognised an abundant PrP^res^ product (PrP^res^ #2) in TgOvPrP4 mice infected with “CH1641-like” isolates, the unglycosylated form of which migrates at ∼14 kDa, in addition to the usual PrP^res^ product (PrP^res^ #1) which migrates at ∼19 kDa in its unglycosylated form. The presence of mono- and di-glycosylated forms derived from this PrP^res^ cleavage product was confirmed by differential immunoprecipitation of PrP^res^ #1 and PrP^res^ #2. Depletion of PrP^res^ #1 using N-terminal antibodies allowed the samples to be enriched in C-terminally cleaved PrP^res^ #2, which then appeared in a 3-band pattern between 14 and 22 kDa. Such experiments also confirm that PrP^res^ #2 is only faintly recognized by Sha 31 antibody, which recognizes the 148–155 region of the ovine PrP protein, suggesting that this region is absent from most of the PrP^res^ #2 fragments. PNGase deglycosylation also facilitated the identification of PrP^res^ #2, and permitted quantification of the respective proportions of PrP^res^ #2 and PrP^res^ #1. Whereas PrP^res^ #2 was abundant in TgOvPrP4 mice infected with “CH1641-like” isolates, lower levels of PrP^res^ #2 could also be detected from 5 natural isolates with h-type PrP^res^ transmitted into TgOvPrP4 mice. C-terminally cleaved PrP^res^ products have previously been described in sporadic or genetic Creutzfeldt-Jakob disease in humans [2]. Although the presence of low levels of PrP^res^ #2 in BSE and L-type BSE cannot be fully excluded, this PrP^res^ form remained undetected in our experiments with these BSE forms, even after differential immunoprecipitation. This was also the case in classical BSE transmitted in a variety of different species. Interestingly, similar results were obtained in TgOvPrP4 mice infected with an isolate from cattle experimentally infected with transmissible mink encephalopathy (TME), consistent with previous studies showing similarities with L-type BSE [29]. Our results thus reinforce the molecular discrimination of “CH1641-like” scrapie isolates from classical BSE, but also indicate a clear molecular difference with L-type BSE transmitted from cattle to ovine transgenic mice. However, further comparisons including those of biological and histopathological features during serial passages in this mouse model will be required, as well as transmission studies performed from L-type BSE experimentally transmitted to sheep.

We have also recently described the identification of a C-terminally cleaved PrP^res^ #2 form in H-type BSE, in cattle and after transmission to C57Bl/6 mice [3]. However, a relationship between “CH1641-like” scrapie isolates and H-type BSE seems unlikely. H-type BSE is indeed characterized by a high PrP^res^ molecular mass comparable to most natural scrapie cases, in contrast to the low PrP^res^ molecular mass, which is the hallmark of “CH1641-like” isolates. Although the transmission of H-type BSE in sheep has not yet been reported, a high PrP^res^ molecular mass was maintained upon transmission in tg338 ovine transgenic mice [26]. Unfortunately, direct comparisons with H-type BSE in TgOvPrP4 mice were not possible since we were unable to transmit the disease from several cattle H-type isolates to these mice, at least at first passage [29]. As these same H-type isolates were transmitted in tg338 expressing higher levels of the V_136_ R_154_ Q_171_ ovine PrP protein [26], this could suggest a high species and/or strain barrier for H-type BSE in sheep. Conversely, both classical and L-type BSEs were readily transmitted in TgOvPrP4 mice [19],[29].

The presence of PrP^res^ #2 within the different scrapie sources, was preferentially associated with PrP^res^ #1 of low molecular mass. When several experimental scrapie sources were analysed, PrP^res^ #2 was only detected in the 87V strain, characterized by l-type PrP^res^, but not in C506M3, Chandler or 79A strains or in the SSBP/1 isolate with h-type PrP^res^, still emphasizing the need of further comparisons between 87V and “CH1641-like” isolates [20]. Although PrP^res^ #2 could also be detected after the transmission of natural scrapie isolates with high molecular mass, the levels were consistently lower than in “CH1641-like” isolates. It might be that the presence of low levels of PrP^res^ #2 in scrapie isolates with h-type PrP^res^ indicates a mixture of PrP^res^ phenotypes in these scrapie sources, with the levels of l-type PrP^res^ undetectable. This possibility should be considered in the light of certain observations. (1) A scrapie case with both h-type and l-type PrP^res^ has recently been described in the UK, each PrP^res^ phenotype originating from two different brain areas [14]. (2) Our recent transmission studies of two “CH1641-like” isolates (O100 and O104) from the same flock into TgOvPrP4 showed the presence of h-type PrP^res^ in some of the mice suggesting a possible mixture of the two PrP^res^ phenotypes in the initial ovine scrapie isolates; these two PrP^res^ phenotypes might be selected, at least in part, during the second passage in TgOvPrP4 mice [20]. Studies of the initial ovine brain samples by immunohistochemistry indeed revealed the presence of differently cleaved PrP^res^ forms in different brain nuclei [13]. (3) Transmission of scrapie in cattle from a brain pool (British source) with h-type PrP^res^ produced two cows with l-typePrP^res^ [36]. h-type PrP^res^ was detected in a second brain sample from one of the two animals. (4) Similar results were observed in a bovine transgenic mouse line, the mobility in mice being faster than in the original scrapie isolate (Irish source) [37]. All together, these data suggest that l-type PrP^res^ could be present in a number of scrapie sources. The identification of “CH1641-like” isolates might be the fortuitous and rare result of analysing samples in which the l-type PrP^res^ of low molecular mass is more abundant.

Further characterization of the biological properties of scrapie sources with l-type PrP^res^ will be required firstly to establish whether these correspond to a single strain of infectious agent or involve a variety of distinct scrapie strains, and secondly to better understand the characteristics of their transmission.

## Materials and Methods

### TSE sources

The TSE sheep isolates (Table 1) included the experimental CH1641 scrapie isolate (kindly provided by N. Hunter, Institute for Animal Health, Edinburgh) and four natural French “CH1641-like” TSE isolates. Transmission studies and initial data concerning the molecular analyses of CH1641 and of two natural “CH1641-like” isolates (O104, TR316211) transmitted to TgOvPrP4 ovine transgenic mice, have already been described [19],[20]. Two other field isolates from A_136_ R_154_ Q_171_ homozygous sheep (05-825 and 06-017) were now included, that showed a low apparent molecular mass of unglycosylated PrP^res^ (0.1–0.4 kDa lower than in cattle BSE), as also described in experimental ovine BSE and reduced PrP^res^ labelling with P4 monoclonal antibody in comparison to most natural scrapie cases that show PrP^res^ of higher molecular mass.

**Table 1:** Breeds and *prnp* genotypes of ovine scrapie sources and survival periods after transmission in TgOvPrP4 ovine transgenic mice.

	Breed	PrP Genotype*	Survival Periods, mean±SD (d.p.i.)**
CH1641	Cheviot	AxQ/AxQ	245±17 (12)
**“CH1641-like” natural isolates**			
O104	Manech tête rousse	VRQ/VRQ	248±50 (10)
TR316211	Unknown	ARQ/ARQ	235±26 (8)
05-825	Unknown	ARQ/ARQ	223±25 (6)
06-017	Unknown	ARQ/ARQ	239±30 (10)
**Other natural scrapie isolates**			
O56	Manech tête rousse	ARH/ARH	392±160 (9)
O69	Manech tête rousse	ARQ/ARQ	252±16 (14)
O87	Manech tête rousse	VRQ/ARQ	353±144 (6)
O111	Unknown	ARQ/ARQ	296±20 (11)
O171	Préalpes du Sud	ARQ/ARQ	247±14 (9)

*: Amino acids 136, 154, and 171.

**: Number of animals.

Other TSE sources examined in TgOvPrP4 mice included (i) 5 natural scrapie isolates identified by clinical surveillance in France, with PrP^res^ of high apparent molecular mass (“non-CH1641-like”) (Table 1); (ii) the SSBP/1 experimental scrapie isolate [19]; (iii) experimental scrapie strains, derived from mouse-adapted strains, C506M3, Chandler, 79A, 87V [19],[20]; (iv) BSE from cattle or obtained after natural transmission (goat, cheetah) [6],[18],[38] or experimental transmission (sheep homozygous for the A_136_R_154_Q_171_ or A_136_R_154_R_171_ *prnp* allele, wild-type mouse)[19], [39]–[41]; and (v) an experimental bovine isolate of transmissible mink encephalopathy [29].

Breeds and *prnp* genotypes of sheep and the survival periods observed after transmission in TgOvPrP4 ovine transgenic mice are shown in Table 1.

### Transmission studies

Four- to six-week-old female TgOvPrP4 ovine transgenic mice [42] were inoculated intra-cerebrally with 10% (first passage) or 1% (second passage) (wt/vol) brain homogenates in 5% glucose in distilled water (20 μl per animal). The brains were sampled at the terminal stage of the disease or death of the animal due to intercurrent disease or ageing. The guidelines of the French Ethical Committee (decree 87–848) and European Community Directive 86/609/EEC regarding mice were respected. Experiments were performed in the Biohazard prevention area (A3) of the author’s institution with the approval of the Rhône-Alpes Ethical Committee for Animal Experiments. The whole brain of every second mouse was frozen and stored at −80°C before Western Blot analysis. The other brains were fixed in 10% formol-saline solution for histopathological examinations.

### Histological examinations

Post-fixed brain were routinely embedded in paraffin after a 1 hour formic acid (98%–100%) treatment. De-waxed and re-hydrated 5 μm brain sections were then either stained using hematoxylin-eosin in order to study vacuolar lesions or immunostained for PrP^sc^ using SAF84 (SPI Bio) and 2G11 (Pourquier) monoclonal antibodies with or without an additional step using streptomycin sulfate, following a procedure reported in detail elsewhere [43]. A peroxydase-labeled avidin-biotin complex (Vectastain Elite ABC, Vector Laboratories) was used to amplify the signal. Final detection was achieved using a solution of diaminobenzidine intensified with nickel chloride (Zymed), producing black deposits. Finally, slides counterstained with aequous hematoxylin were observed under a microscope coupled to an image analysis workstation (Morpho Expert software, ExploraNova). The lesion profiles were built following referential criteria [44] using a computer-assisted method [45].

### Extraction of PrP^res^

PrP^res^ was obtained following concentration by ultra-centrifugation from half of the mouse brains homogenised in glucose 5% in distilled water (20% wt/vol). A 600 μl volume was made up to 1.2 ml in glucose 5%, before incubation with proteinase K (10 μg/100 mg brain tissue) (Roche) for 1 h at 37°C. N-lauroyl sarcosyl 30% (600 μl; Sigma) was added. After incubation at room temperature for 15 min, samples were then centrifuged at 100,000 rpm for 2 h on a 400 μl 10% sucrose cushion, in a Beckman TL100 ultracentrifuge. Pellets were resuspended and heated for 5 min at 100°C in 50 μl TD4215 denaturing buffer (SDS 4%, β-mercaptoethanol 2%, glycine 192 mM, Tris 25 mM, sucrose 5%). In some experiments, deglycosylation was performed using PNGase F (kit P07043, BioLabs), as previously described [20].

### Differential immunoprecipitation

Differential immunoprecipitation was used to enrich the samples in the C-terminally cleaved form of PrP^res^ (PrP^res^ #2), by depletion of the usual form of PrP^res^ (PrP^res^ #1).

Superparamagnetic polystyrene beads coated with a monoclonal antibody specific for Fc on all mouse IgG (Dynabeads® Pan Mouse IgG_DYNAL #110.41) were used as recommended by the manufacturer. After each step, the beads were recovered using Dynal PMC. 50 μl bead aliquots were washed 3 times in 5 volumes of coating buffer (PBS with 0.1% of BSA). Beads (50 μl of beads resuspended in 50 μl coating buffer) collected after the last washing were then coated with IgG mouse monoclonal antibodies Sha 31 or SAF84 (ascitic fluids; SPI-Bio, France) for PrP^res^ #1 or PrPres #2 capture, respectively. Sha31 and SAF84 recognise the ovine PrP sequences 148-YEDRYYRE-155 and 167-RPVDQY-172, respectively. For each cycle of PrP^res^ capture, the sample was incubated with antibody-coated beads for 1 h at room temperature under continuous rotation at 60 rpm.

After PrP^res^ ultracentrifugation, the pellets obtained from 2 mg brain tissues were resuspended in 20 μl immunoprecipitation buffer (phosphate-buffered saline [PBS] at pH 7.4 and 0.3% of N-lauroyl sarcosyl) and heated 5 min at 100°C, before addition of a 30 μl suspension of antibody-coated beads. After completing the beads suspension to 1 ml, the sample was enriched in PrP^res^ #2, by depleting the PrP^res^ #1 in 5 to 7 successive rounds of PrP^res^ #1 capture using Sha31-coated beads. The supernatants collected after each capture cycle were used for the next one. PrP^res^ #2 was then captured by SAF84-coated beads. At the end of each capture cycle, PrP^res^ was removed from the beads by heat denaturation for 5 min at 100°C in 30 μl TD4215 buffer prior to Western blot analyses.

### Western blot analyses

Western blot analysis was performed as previously described [20] by 15% SDS-PAGE and electroblotting on nitrocellulose membranes. PrP^res^ was detected with P4 (0.2 μg/ml) (93-WGQGGSH-99 ovine PrPsequence; R-Biopharm, Germany), Bar233 (1/5000) (144-FGNDYEDRYYRE-155 ovine PrP sequence; kindly provided by J. Grassi, C.E.A.-Saclay, France), Sha31 (1/10 from TeSeE Bio-Rad sheep and goats kit; Bio-Rad, France) or SAF84 (SPI-Bio, France) mouse monoclonal antibodies. Peroxidase-labelled conjugate anti-mouse IgG (H+L) (1/2500 in PBST; ref 1010-05; Clinisciences, France) was used to detect P4, Bar233, and Sha31 antibodies, whereas SAF84 was used as horseradish peroxidase antibody. Streptavidin (5 ng/ml) (S5512) was added to the conjugate solution. Bound antibodies were then detected by direct capture with the Versa Doc (Bio-Rad) analysis system using the ECL chemiluminescent substrate (Amersham, France). Quantitative studies were performed using Quantity One (Bio-Rad) software, and the apparent molecular masses were evaluated by comparing the positions of the PrP^res^ bands with a biotinylated marker (B2787) (Sigma, France).

### Statistical analyses

The glycoforms proportions of the four “CH1641-like” isolates and the CH1641 isolate were compared with each other and the glycoforms proportions of both classical BSE and L-type BSE were compared with those of each natural “CH1641-like” isolate and with the experimental CH1641 isolate. Comparison of classical BSE alone with each “CH1641-like” isolate and with CH1641 at first passage implies 5 tests for each of the 3 PrP^res^ #1 bands. In view of the high total number of tests (19 for each PrP^res^ band), paired-sample *t* tests with Bonferroni adjustment were used to preclude the detection of spurious differences in glycoform proportions.

A classical analysis of variance was used for comparisons of PrP^res^ #2. The statistical analysis was performed with R software (R version 2–6.0 [2007-11-03]: A language and environment for statistical computing. R Foundation for Statistical Computing, Vienna, Austria. ISBN 3-900051-07-0; http://www.R-project.org).

## Supporting Information

Figure S1 Western blot comparisons of PrP^res^ in the brains of TgOvPrP4 mice and sheep or cattle. PrP^res^ from sheep (lanes 1, 5, and 7) or cattle (lane 3) and from TgOvPrP4 mice (lanes 2, 4, 6, and 8) was detected using Bar233 (A) and SAF84 (B) antibodies. TSE sources were CH1641, BSE-L, 06-017 (“CH1641-like”), and BSE-C.
(2341 KB TIF)

Figure S2 Neuropathological features of “CH1641-like” isolates in TgOvPrP4 mice. (A-D) Brain lesion profiles (left panels) and disease-associated prion protein brain mapping (right panels) observed in the brains of TgOvPrP4 mice (n = 3–7) infected at first passage with O104, TR316211, 05-825, or 06-017 isolates. 1. Dorsal medulla nuclei. 2. Cerebellar cortex. 3. Superior colliculus. 4. Hypothalamus. 5. Central thalamus. 6. Hippocampus. 7. Lateral septal nuclei. 8. Cerebral cortex at the level of thalamus. 9. Cerebral cortex at the level of septal nuclei. (E) Immunohistochemical detection of disease-associated prion protein in the brain of TgOvPrP4 mice; signal intensity was different in the cortex between L-type BSE and “CH1641-like” sheep scrapie. In the raphe dorsalis intensity was similar, but the deposition was slightly different and appeared intraneuronal in the case of the “CH1641-like” sheep scrapie group.
(0.88 MB TIF)
