# Supplementary figures and images for: A C-Terminal Protease-Resistant Prion Fragment Distinguishes Ovine “CH1641-Like” Scrapie from Bovine Classical and L-Type BSE in Ovine Transgenic Mice
Source: PLoS Pathog. 2008 Aug 29;4(8):e1000137. doi: 10.1371/journal.ppat.1000137 (PMC2516186; doi:10.1371/journal.ppat.1000137)

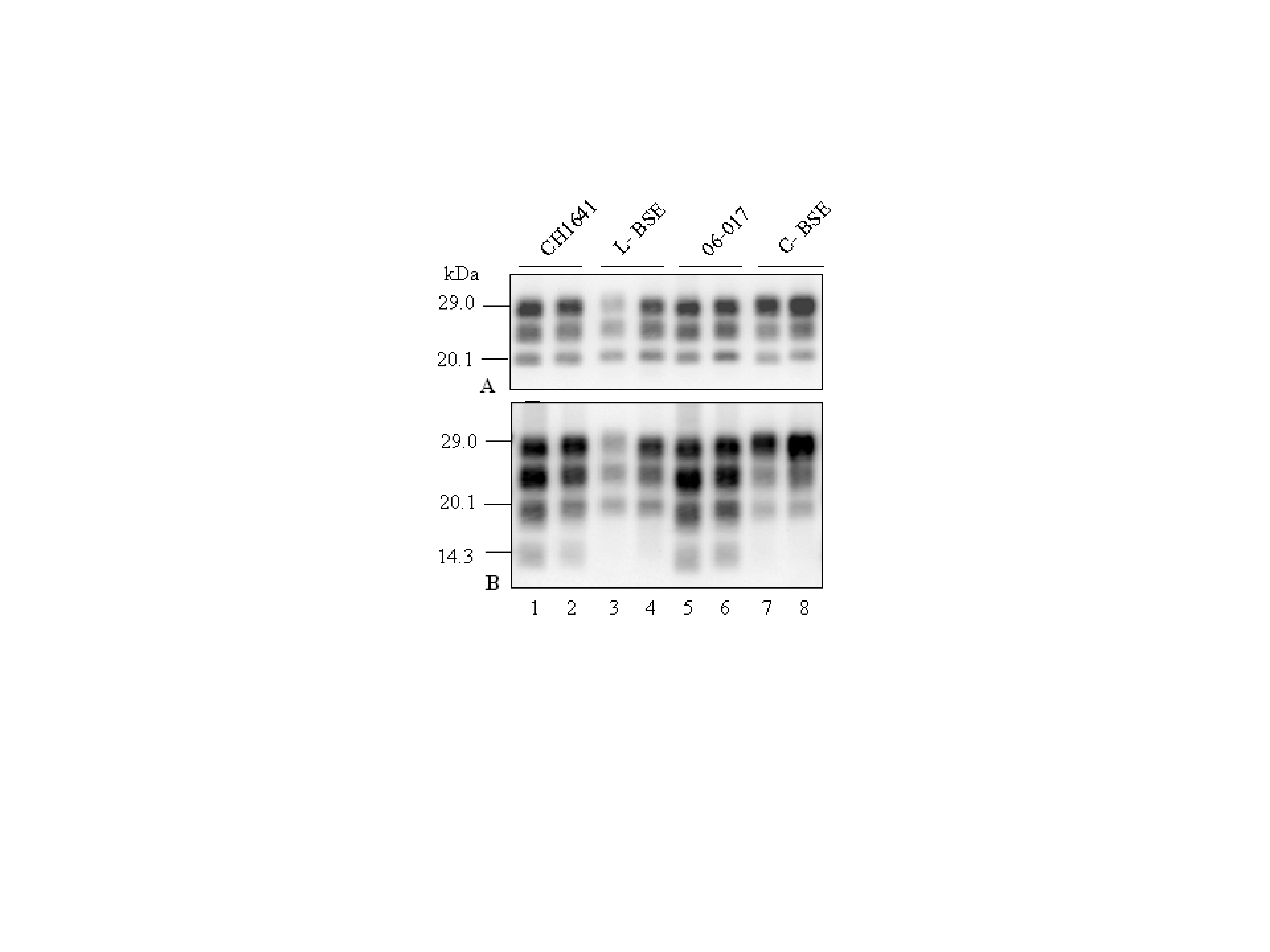

Supplement: Figure S1 — Western blot comparisons of PrPres in the brains of TgOvPrP4 mice and sheep or cattle. PrPres from sheep (lanes 1, 5, and 7) or cattle (lane 3) and from TgOvPrP4 mice (lanes 2, 4, 6, and 8) was detected using Bar233 (A) and SAF84 (B) antibodies. TSE sources were CH1641, BSE-L, 06-017 (“CH1641-like”), and BSE-C. (2341 KB TIF) [file ppat.1000137.s001.tif]

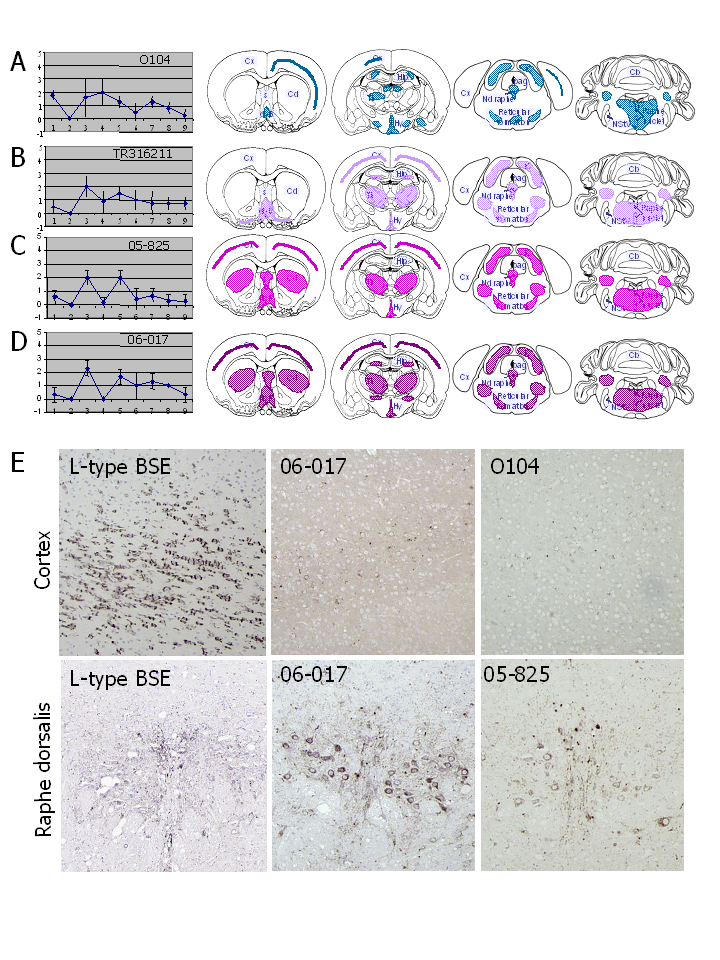

Supplement: Figure S2 — Neuropathological features of “CH1641-like” isolates in TgOvPrP4 mice. (A-D) Brain lesion profiles (left panels) and disease-associated prion protein brain mapping (right panels) observed in the brains of TgOvPrP4 mice (n = 3–7) infected at first passage with O104, TR316211, 05-825, or 06-017 isolates. 1. Dorsal medulla nuclei. 2. Cerebellar cortex. 3. Superior colliculus. 4. Hypothalamus. 5. Central thalamus. 6. Hippocampus. 7. Lateral septal nuclei. 8. Cerebral cortex at the level of thalamus. 9. Cerebral cortex at the level of septal nuclei. (E) Immunohistochemical detection of disease-associated prion protein in the brain of TgOvPrP4 mice; signal intensity was different in the cortex between L-type BSE and “CH1641-like” sheep scrapie. In the raphe dorsalis intensity was similar, but the deposition was slightly different and appeared intraneuronal in the case of the “CH1641-like” sheep scrapie group. (0.88 MB TIF) [file ppat.1000137.s002.tif]
